# Associations between folate metabolism biomarkers and cognitive impairment in older Chinese adults: a cross-sectional study

**DOI:** 10.3389/fmed.2025.1522531

**Published:** 2025-08-18

**Authors:** Huimin Lv, Jia Li, Lu Chen, Keyi Lu, Xudong Zhao, Mengyuan Guo, Hailong Lu

**Affiliations:** ^1^Department of Geriatrics, The Affiliated Hospital of Xuzhou Medical University, Xuzhou, Jiangsu, China; ^2^Department of General Medicine, The Affiliated Hospital of Xuzhou Medical University, Xuzhou, Jiangsu, China

**Keywords:** vitamin B2, S-adenosylmethionine, homocysteine, folate metabolism, age-related cognitive impairment, cross-sectional study

## Abstract

**Background:**

The role of folate metabolism–related biomarker profiles in age-related cognitive impairment (ARCI) remains unclear. This cross-sectional study aimed to examine the association between folate metabolism-related biomarkers and cognitive performance in older Chinese adults.

**Methods:**

We conducted a cross-sectional analysis of 100 participants aged between 56 and 87 years. Cognitive status was classified as ARCI if participants met the cutoff criteria on both the MMSE (<27) and MoCA (<26). Those meeting the cutoff criteria on both MMSE (≥27) and MoCA (≥26) were classified as cognitively normal (CN). Serum levels of folate metabolism-related biomarkers were compared between groups and analyzed for their associations with cognitive scores. Logistic regression was used to examine associations between individual biomarkers and cognitive impairment status. Multiple linear regression analyses were conducted to assess relationships with MMSE and MoCA scores, adjusting for age and education.

**Results:**

Cognitive impairment was prevalent among older adults at a rate of 56.3% (*P* < 0.05). The ARCI group showed significantly lower levels of vitamin B2 (VB2), folate (VB9) and lower MMSE and MoCA scores compared to the CN, while S-adenosylhomocysteine (SAH) and homocysteine (Hcy) levels were higher. MMSE and MoCA scores were positively correlated with serum VB2, VB9, and plasma S-adenosylmethionine (SAM) levels and negatively correlated with Hcy levels. Logistic regression showed that education and serum Hcy were significantly associated with cognitive impairment (AUC = 0.73). In addition to age, VB2, SAM and Hcy in the folate metabolic profile were significantly associated with MMSE and MoCA scores, accounting for 45.9 and 42.7% of the variance in these scores, respectively.

**Conclusions:**

VB2, SAM and Hcy may be associated with cognitive impairment in older Chinese adults and warrant further investigation as potential biomarkers.

## 1 Introduction

China is facing an increasingly severe challenge of population aging. According to national projections, the number of Chinese citizens aged 60 and above is expected to reach 254 million in 2019 and exceed 402 million by 2040, accounting for ~28% of the total population (1). This dramatic shift poses pressing public health challenges, particularly in managing chronic conditions and geriatric syndromes. Geriatric syndromes refer to a condition that significantly impacts the quality of life in older adults due to the gradual decline in bodily functions associated with aging and the presence of multiple chronic diseases (2).

Among the various manifestations of geriatric syndromes, age-related cognitive impairment (ARCI) stands out due to its high prevalence and the growing demands it places on long-term care and healthcare systems. The concept of ARCI was first proposed by the International Psychogeriatric Association in 1994 to describe cognitive deterioration stemming from age-related dysregulation in neural networks (3). ARCI encompasses impairments in domains such as perception, attention, memory, language, or executive function, significantly interfering with daily and social functioning, yet do not meet the diagnostic threshold for dementia. ARCI represents a spectrum of cognitive dysfunction that differs from dementia in severity but is nonetheless clinically significant. In recent years, the term ARCI has been increasingly adopted in geriatric and cognitive aging research. It is used to describe early cognitive decline that does not meet criteria for dementia, as reported in both clinical and animal model studies (4–6). Epidemiological studies estimate that ~10%−20% of older adults experience mild cognitive impairment or eventually develop dementia (7). Cognitive impairment significantly compromises self-care capacity and quality of life in older adults. It is also associated with increased hospitalization rates, elevated mortality risk, and heightened social care expenditures, thereby imposing a substantial economic burden on society (8–10). Thus, early and accurate identification of cognitive impairment is essential to enable timely intervention, slow disease progression, and mitigate both familial and societal burdens.

Recent advances in folate metabolic research have highlighted its mechanistic relevance to cognitive function, offering novel perspectives for early diagnosis and pathophysiological understanding of cognitive impairment. The folate metabolic pathway involves the conversion of homocysteine (Hcy) to S-adenosylmethionine (SAM) in the presence of vitamin B12 (VB12), supplying methyl groups essential for the central nervous system. During methyl transfer, SAM is converted to S-adenosylhomocysteine (SAH), which is subsequently broken down into Hcy. vitamins B2 (VB2) and B6 (VB6) act as coenzymes in these reactions, supporting the smooth transfer of one-carbon units and ensuring proper methylation in neurotransmitter synthesis (11, 12). This process plays a crucial role in regulating cognitive functions. Disruptions in this cycle may lead to elevated Hcy, reduced SAM levels, and impaired methylation capacity, all of which have been implicated in cognitive impairment (13).

Despite growing mechanistic evidence, gaps remain in the clinical application of folate-related biomarkers in cognitive assessment. Previous studies have consistently identified associations between low levels of serum folate or B vitamins and poorer cognitive outcomes, with clinical evidence indicating that supplementation with B vitamins may enhance cognitive performance and mitigate brain atrophy (14, 15). In a large cohort of older Chinese adults, Chen et al. (16) found that higher plasma folate levels were associated with a 59% reduction in the risk of cognitive impairment. However, the relationship between folate metabolism and cognitive impairment remains unclear, partly due to the lack of studies investigating multiple folate-related biomarkers simultaneously. Most previous research has focused on single nutrients in isolation, without considering their interactive roles within the metabolic pathway, particularly in older Chinese adults (12, 13, 16).

Therefore, this cross-sectional study aimed to estimate the prevalence of cognitive impairment among older Chinese adults, compare folate metabolism–related biomarker levels across cognitive status groups, and examine the cross-sectional associations between these biomarkers and cognitive performance.

## 2 Methods

### 2.1 Human participants

One hundred patients, aged 56–87 years, were hospitalized in the Geriatrics Department of the Affiliated Hospital of Xuzhou Medical University (XZMU) and divided into two groups based on their Mini-Mental State Examination (MMSE) and Montreal Cognitive Assessment (MoCA) scores. Among them, 71 individuals aged 65 years or older were selected for analysis. Inclusion criteria: (1) good mental status, able to cooperate with medical instructions and complete assessments; (2) informed consent signed by the patient or their legal representative. Exclusion criteria: (1) neurological diseases affecting cognitive function, including Alzheimer's disease (AD), Parkinson's disease (PD), vascular dementia, stroke, epilepsy, or central nervous system infections; (2) history of psychiatric disorders, such as schizophrenia or depression; (3) previous cerebrovascular diseases, including transient ischemic attack, stroke, or intracranial hemorrhage; (4) serious infections, tumors, anemia, or history of medications affecting cognition, including benzodiazepines, antidepressants, and anxiolytics; (5) alcoholic encephalopathy. This study was reviewed and approved by the Ethics Review Committee of the Affiliated Hospital of Xuzhou Medical University (XYFY2022-KL053-01).

### 2.2 Blood collection and biochemical measurements

Scales were used to collect demographic information, including gender, age, and years of education. Physical measurements such as weight and height were obtained using a standard balance beam scale, and body mass index (BMI) was calculated as weight (kg) divided by height squared (m^2^). Blood samples were collected between 7:00 and 8:00 a.m. after an overnight fast. Routine biochemical parameters (e.g: AST, ALT, GGT, ALP, total protein, albumin, bilirubin, glucose, and lipids) were measured using standard automated analyzers (BS-2000M, Mindray, China) as part of the baseline clinical assessment. Tau-181 and Aβ1-42 were quantified using ELISA kits from Shuangying Biotechnology Co., Ltd. (Shanghai, China). All biomarker assays were conducted in a blinded manner, and batch effects were minimized by analyzing all samples in a single run.

### 2.3 Folate metabolic profiles test

In this study, blood samples were collected after participants fasted from food and water beginning at 10:00 p.m. on the day of admission. Venous blood was drawn from the median vein between 7:00 and 8:00 a.m. the following morning. Two tubes of blood were collected from each subject: 3 ml in a procoagulant tube and 3 ml in an EDTA-K2 anticoagulant tube. The samples were gently mixed and allowed to stand for 30 min before centrifugation at 5,000 rpm for 15 min. Serum and plasma were separated and transferred into 1.5 ml EP tubes, which were then stored at −80°C for batch analysis. Folate metabolism–related biomarkers were subsequently analyzed using an ACQUITY ultra-performance liquid chromatography (UPLC) system coupled with a Xevo TQ-S triple quadrupole mass spectrometer (Waters Corporation, USA).

### 2.4 Cognitive function assessment and classification

In a quiet, distraction-free environment, two specialized neurologists assessed cognitive function using the Mini-Mental State Examination (MMSE) and the Montreal Cognitive Assessment (MoCA). The MMSE evaluates orientation, memory, attention and calculation, recall, and language abilities (naming, repetition, command following, reading, writing, and figure drawing), while the MoCA covers short-term memory, visuospatial skills, executive functioning, attention, working memory, language, and temporal orientation. Both tests are scored out of 30 points. For MoCA, one additional point was added for participants with 12 or fewer years of education to adjust for educational background.

Participants were classified into two groups based on their performance: those meeting the cutoff criteria on both MMSE (<27) and MoCA (<26) were classified as having ARCI, while those meeting the cutoff criteria on both MMSE (≥27) and MoCA (≥26) were classified as cognitively normal (CN). The MMSE cutoff of <27 was selected based on Mitchell's meta-analysis, which showed improved sensitivity for detecting MCI compared to the conventional threshold (17). Although validation studies using this MMSE threshold in mainland China are limited, this threshold has been adopted in East Asian populations with similar socio-cultural and educational contexts (18). The MoCA cutoff of <26 was derived from its original validation study, which demonstrated 90% sensitivity and 87% specificity for detecting mild cognitive impairment (19), and has further been validated in Chinese older adults with excellent diagnostic performance (20). Combining both criteria may help improve the robustness and diagnostic performance of cognitive screening.

### 2.5 Statistical analysis

Statistical analyses were performed using JASP 0.19.1. No imputation was performed for missing data. Analyses were based on complete cases only. Measurement data that followed a normal distribution were described as mean ± standard deviation (x ± s), and comparisons between groups were made using the independent samples *t*-test. For data that did not follow a normal distribution, the median and interquartile range [M (P25, P75)] were used, with comparisons between groups made using the Mann-Whitney U-test. Categorical data were expressed as percentages (%), and intergroup comparisons were performed using the chi-square (χ^2^) test. Pearson's correlation analysis was employed to evaluate the relationship between MMSE and MoCA scores and folate metabolic profiles in older adults. Statistical significance was set at *P* < 0.05. The variance in cognitive function explained by folate metabolic profiles was determined using stratified multiple regression, accounting for age and education. Due to the relatively small sample size, comorbidities and medication use were not included as covariates. However, individuals with neurological, psychiatric disorders or medications known to affect cognition were excluded at enrollment to minimize potential confounding. Given the exploratory nature of this study, adjustments for multiple comparisons were not performed.

## 3 Results

### 3.1 Comparison of the prevalence of cognitive impairment in different age groups

A total of 100 participants were included in this study, of whom 29 were younger than 65 years and 71 were 65 years or older. The prevalence of cognitive impairment was significantly higher in the elderly group (56.3%) compared to the non-elderly group (20.6%) (χ^2^ = 10.534, *P* = 0.0025).

### 3.2 Baseline characteristics of the two groups

A total of 31 cognitively normal (CN) participants and 40 ARCI participants were included in the study. The general and clinical characteristics of the elderly participants are presented in Table 1. Statistically significant differences were observed between the two groups in terms of age, years of education, total bilirubin, direct bilirubin, and total protein levels (*P* < 0.05). The MMSE and MoCA scores of the ARCI group were significantly lower than those of the CN group, and the differences were statistically significant (*P* < 0.001). There were no significant differences between the two groups in other baseline biochemical markers, including AST, ALT, GGT, lipid profiles, Tau-181, and Aβ1-42 (all *P* > 0.05).

**Table 1:** Characteristics of the HC and ARCI participants in this study.

**Characteristic**	**CN (*n* = 31)**	**ARCI (*n* = 40)**	***Z*/*t*/χ^2^**	***P***
Age (years)	70 (67, 72)	72 (68.25, 76)	−2.106	**0.035** ^*^
Sex			0.848	0.357
Male	15 (48.40%)	15 (37.50%)		
Female	16 (51.60%)	25 (62.50%)		
AST (U/L)	17 (14, 21)	19 (16.25, 22)	−1.633	0.102
ALT (U/L)	13 (10, 26)	13 (9.25, 18.75)	−0.627	0.53
GGT (U/L)	21 (14, 31)	14.5 (12, 22.75)	−1.532	0.125
Total bilirubin (μmol/L)	12.8 (10.2, 16.8)	9.95 (7.75, 13.23)	−2.435	**0.015** ^*^
Direct bilirubin (μmol/L)	4.7 (4, 6)	3.7 (3.05, 4.97)	−2.407	**0.016** ^*^
Total bile acids (μmol/L)	4.1 (2.1, 7.3)	5.9 (3.2, 9.23)	−1.618	0.106
Fasting glucose (mmol/L)	5.26 (4.7, 6.48)	5.36 (4.53, 6.28)	−0.157	0.876
Triglyceride (mmol/L)	1.08 (0.79, 1.41)	1.05 (0.77, 1.51)	−0.226	0.821
TSH (mIU/L)	2.09 (1.45, 3.57)	1.9 (1.46, 3.22)	−0.081	0.935
TAU-181 (ng/ml)	18.1 (14.4, 28.8)	28.7 (11.05, 72.58)	−1.676	0.094
Aβ1-42 (ng/ml)	31.1 (13.7, 85.3)	45.8 (26.88, 104.8)	−1.78	0.075
Glycated hemoglobin (%)	5.9 (5.5, 7.7)	6.1 (5.7, 7.78)	−1.01	0.313
Education level (years)	9 (6, 12)	6 (0, 9)	−2.348	**0.019** ^*^
BMI (kg/m^2^)	24.03 ± 3.41	23.42 ± 3.82	0.701	0.486
ALP (U/L)	74.68 ± 16.81	76.63 ± 19.34	−0.445	0.658
Total protein (g/L)	65.74 ± 5.94	69.77 ± 6.53	−2.679	**0.009** ^**^
Albumin (g/L)	40.43 ± 3.33	41.8 ± 2.88	−1.862	0.067
Creatinine (μmol/L)	57.81 ± 15.25	61.9 ± 14.02	−1.174	0.244
uric acid (μmol/L)	272.87 ± 78.66	261.75 ± 68.6	0.635	0.527
Total cholesterol (mmol/L)	4.18 ± 0.91	4.49 ± 1.19	−1.173	0.245
HDL-C (mmol/L)	1.24 ± 0.33	1.29 ± 0.41	−0.576	0.566
LDL-C (mmol/L)	2.47 ± 0.8	2.72 ± 0.95	−1.177	0.243
MMSE	29.00 (28.00, 29.00)	24.50 (20.00, 26.00)	−7.263	**<0.001** ^***^
MoCA	27.00 (27.00, 28.50)	20.00 (16.00, 25.00)	−7.244	**<0.001** ^***^

The data are expressed as the mean ± standard deviation. For data that did not follow a normal distribution, the median and interquartile range [M (P25, P75)] were used. Categorical data were expressed as percentages (%). CN, cognitively normal; ARCI, age-related cognitive impairment; AST, aspartate aminotransferase; ALT, alanine aminotransferase; GGT, gamma-glutamyl transferase; TSH, thyroid-stimulating hormone; BMI, body mass index; ALP, alkaline phosphatase; HDL-C, high-density lipoprotein cholesterol; LDL-C, low-density lipoprotein cholesterol; MMSE, Mini-Mental State Examination; MoCA, Montreal Cognitive Assessment. ^*^P < 0.05, ^**^P < 0.01, and ^***^P < 0.001. Bold values indicate statistically significant differences (*P* < 0.05).

### 3.3 Comparison of folate metabolic profiles of the two groups

**Figure 1:**
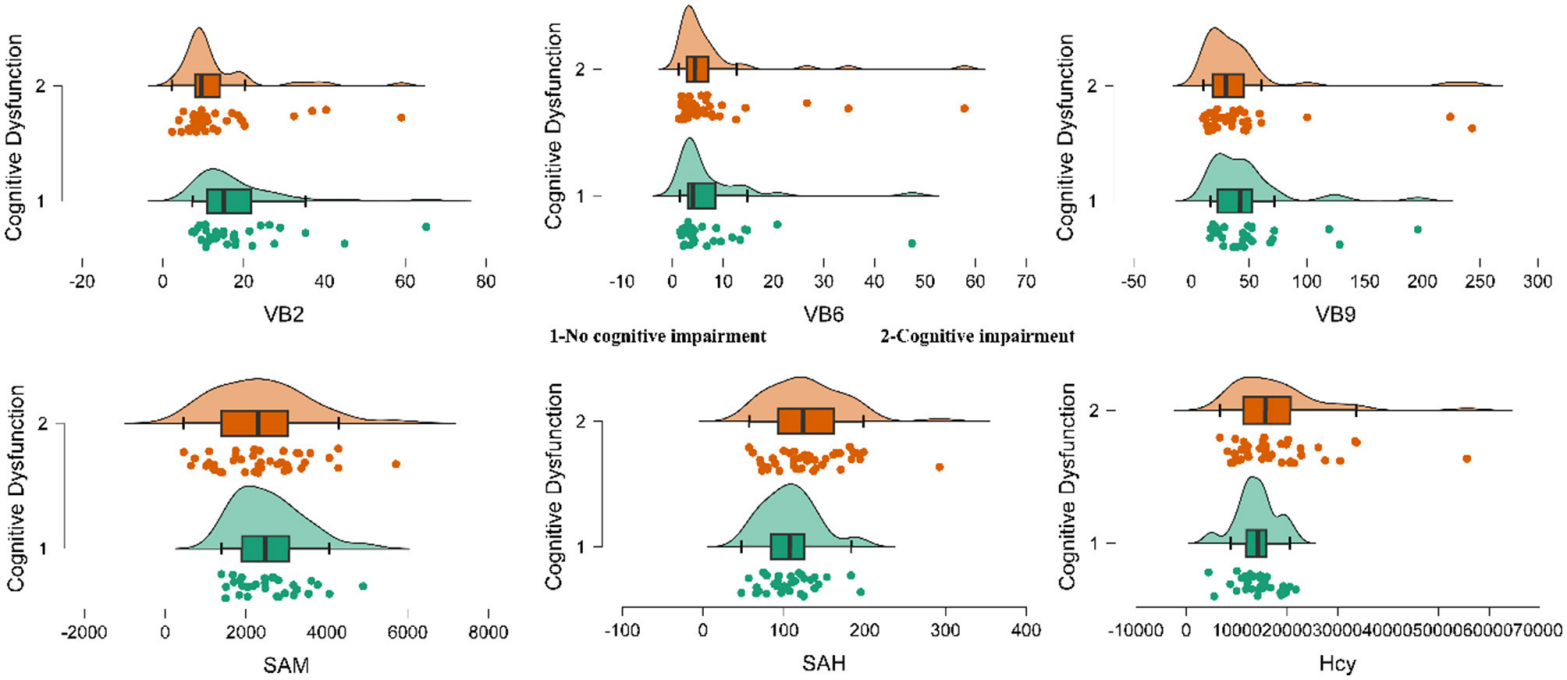
The rainbow plot of the descriptive statistics of the serum levels of folate of the two groups. Group 1 represents participants without cognitive impairment, while Group 2 represents participants with cognitive impairment. Significant differences were observed between the CN and ARCI groups in VB2, VB9, SAH, and HCY levels.

The results showed that the levels of VB2 and VB9 in the ARCI group were significantly lower than those in the control group, while the levels of SAH and Hcy were significantly higher. These differences were statistically significant, as presented in Table 2 and Figure 1.

**Table 2:** Comparison of the serum levels of folate of the HC and ARCI participants.

**Biomarker (ng/ml)**	**CN (*n* = 31)**	**ARCI (*n* = 40)**	***Z/t***	***P***
VB2 (ng/ml)	14.98 (10.89, 21.79)	9.59 (7.99, 14.07)	−3.107	**0.002** ^**^
VB6 (ng/ml)	4.06 (3.12, 8.43)	4.41 (2.85, 7.01)	−0.151	0.880
VB9 (ng/ml)	42.34 (22.63, 52.05)	29.75 (18.38, 45.51)	−1.994	**0.046** ^*^
SAM (ng/ml)	2561.58 ± 837.09	2360.46 ± 1139.46	0.825	0.412
SAH (ng/ml)	107.72 ± 34.41	129.5 ± 48.16	−2.13	**0.037** ^*^
Hcy (ng/ml)	13920.53 ± 4018.65	17662.54 ± 9163.16	−2.119	**0.038** ^*^

The data are expressed as the mean ± standard deviation. For data that did not follow a normal distribution, the median and interquartile range [M (P25, P75)] were used. CN, cognitively normal; ARCI, age-related cognitive impairment; VB2, vitamins b2; VB6, vitamins b6; VB9, vitamins b9 or folate; SAM, S-adenosylmethionine; SAH, S-adenosylhomocysteine; Hcy, homocysteine; ^*^P < 0.05, ^**^P < 0.01. Bold values indicate statistically significant differences (*P* < 0.05).

### 3.4 The correlation effects between MMSE and MoCA scores and folate metabolic profiles in older adults

**Figure 2:**
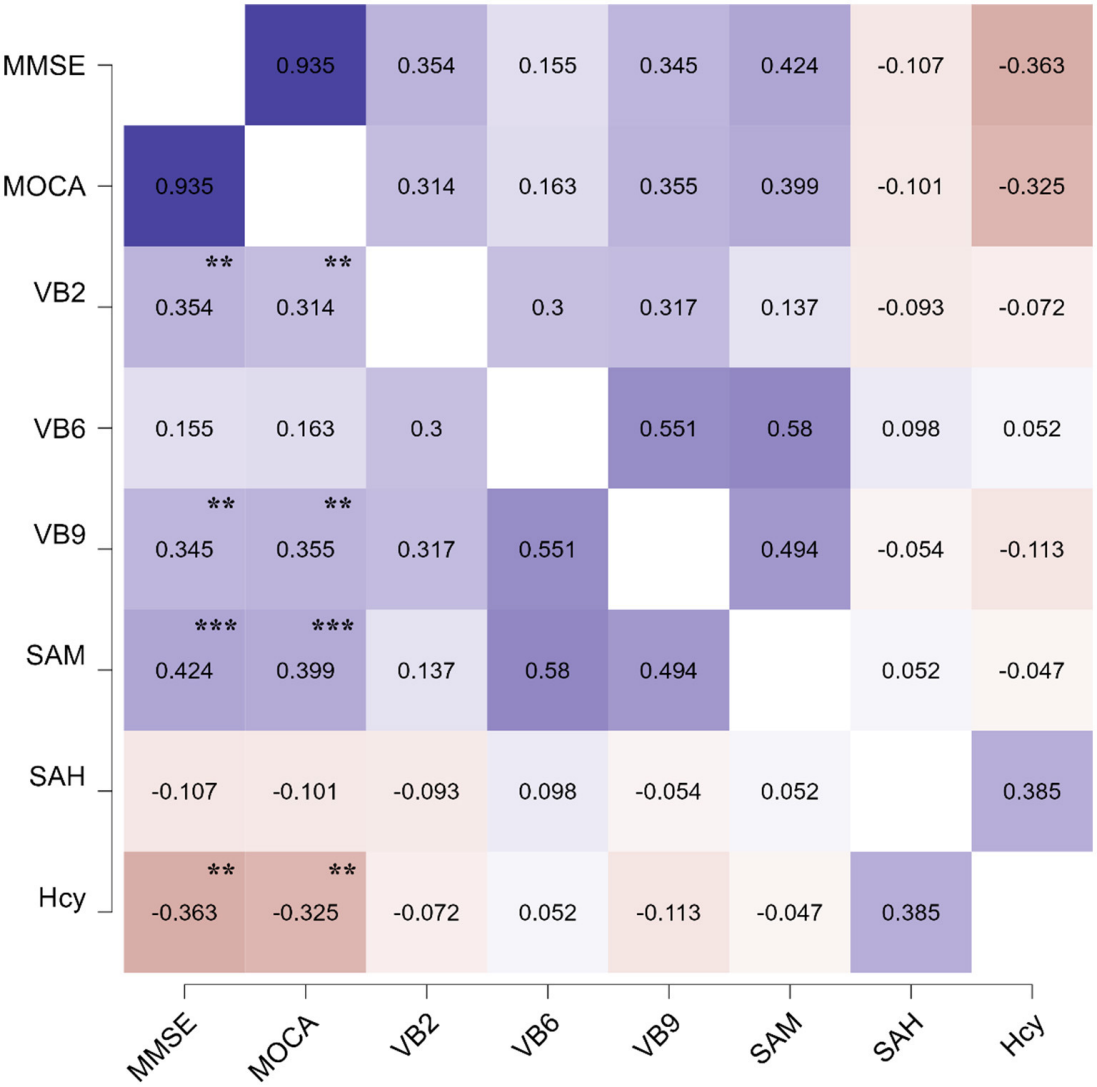
The pearson's heatmap of the correlation effects between the serum levels of folate and MMSE, MoCA scores. The significant of the correlation effects was shown in the figure. ^**^P < 0.01 and ^***^P < 0.001.

To verify the value of folate metabolic profiling in clinical practice, we performed correlation analyses between folate metabolic profiling and cognitive scale scores. Serum VB2 (MMSE: *r* = 0.354, *P* = 0.002; MoCA: *r* = 0.314, *P* = 0.008), VB9 (MMSE: *r* = 0.345, *P* = 0.003; MoCA: *r* = 0.354, *P* = 0.002) and SAM (MMSE: *r* = 0.424, *P* < 0.001; MoCA: *r* = 0.399, *P* < 0.001) levels were positively correlated with MMSE and MoCA scores. In contrast, Hcy was negatively correlated with MMSE (*r* = −0.363, *P* = 0.002) and MoCA scores (*r* = −0.329, *P* = 0.006), as shown in Table 3 and Figure 2.

**Table 3:** The correlation effects between MMSE and MoCA scores and the serum levels of folate.

**Biomarker**	**MMSE**		**MoCA**	
	***r***	***p***	***r***	***p***
VB2	0.354^**^	0.002	0.314^**^	0.008
VB6	0.155	0.158	0.163	0.175
VB9	0.345^**^	0.003	0.355^**^	0.002
SAM	0.424^**^	<0.001	0.399^***^	<0.001
SAH	−0.107	0.375	−0.101	0.401
Hcy	−0.363^**^	0.002	−0.325^**^	0.006

MMSE, Mini-Mental State Examination; MoCA, Montreal Cognitive Assessment; VB2, vitamins b2; VB6, vitamins b6; VB9, vitamins b9 or folate; SAM, S-adenosylmethionine; SAH, S-adenosylhomocysteine; Hcy, homocysteine. ^**^P < 0.01 and ^***^P < 0.001.

### 3.5 Logistic regression analysis of folate metabolic profiles and their association with cognitive impairment

**Figure 3:**
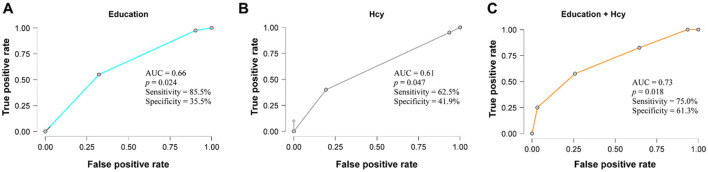
ROC curve plot of the discriminative performance of the Education and Hcy variables for cognitive impairment. **(A)** Education; **(B)** Hcy; **(C)** Combination of Education and Hcy.

Logistic regression analysis indicated that education level and Hcy levels were significantly associated with cognitive impairment. Logistic regression was performed to ascertain the effects of education and Hcy variables on the likelihood that participants have a cognitive dysfunction. Higher education was correlated with a lower likelihood of cognitive impairment (OR = 0.84, 95% CI: 0.74–0.96, *P* = 0.008), while elevated Hcy levels were correlated with a higher likelihood (OR = 1.0003, 95% CI: 1.0000–1.0006, *P* = 0.018). The logistic regression model was statistically significant, χ^2^ (2) = 13.33, *P* = 0.001, with a Nagelkerke's *R*^2^ = 0.229, indicating a moderate model fit. Receiver operating characteristic (ROC) analysis showed an area under the curve (AUC) of 0.66 (95% CI: 0.54–0.78, *P* = 0.024) for education and 0.61 (95% CI: 0.48–0.74, *P* = 0.047) for Hcy. The combined model yielded an AUC of 0.73 (95% CI: 0.62–0.84, *P* = 0.018), indicating acceptable discrimination in distinguishing between cognitively impaired and normal participants (Figure 3).

### 3.6 Linear regression analysis of MMSE and MoCA scores and folate metabolic profiles

As shown in Tables 4, 5, multiple linear regression using the enter method was performed to assess the associations of continuous variables (age, education and folate metabolism-related products) with MMSE and MoCA scores. Previous studies have shown that age and education are associated with cognitive impairment (7, 21). Folate metabolic profile variables were included to evaluate whether they were also associated with MMSE and MoCA scores. In the initial models, age and education explained 11.5 and 13% of the total variance in the MMSE and MoCA, respectively; the explanatory power of the MMSE and MOCA models was significantly improved with the addition of the folate metabolic profile indicator, which increased the variance explained by ~45.9%, *F* (4, 66) = 9.051, *P* < 0.001, *R*^2^ = 0.459, respectively; and 42.7%, *F* (4, 66) = 7.961, *P* < 0.001, *R*^2^ = 0.427, respectively. Each additional year of age was associated with decreases of 0.264 (95% CI: −0.337 to −0.052, *P* = 0.008) and 0.322 points (95% CI: −0.531 to −0.125, *P* = 0.002) in MMSE and MoCA scores, respectively. Higher levels of VB2 and SAM were associated with increases of 0.249 (95% CI: 0.015 to 0.14, *P* = 0.016) and 0.354 (95% CI: 0.001 to 0.002, *P* = 0.001) points in MMSE scores, and 0.212 (95% CI: 0.002 to 0.181, *P* = 0.045) and 0.317 (95% CI: 0.000495 to 0.003, *P* = 0.005) points in MoCA scores, respectively. Additionally, each 1-ng/ml increase in Hcy was associated with a decrease of 0.25 points in MMSE (95% CI: −0.000215 to −0.000027, *P* = 0.012).

**Table 4:** Linear regression for MMSE.

**Model**	**Unstandardized**	**Standard error**	**Standardized**	***t***	***p***
**M**_1_					
(Intercept)	35.717	4.920		7.260	<0.001
Age	−0.194	0.071	−0.264	−2.724	0.008^**^
Education	0.125	0.076	0.157	1.642	0.106
VB2	0.078	0.031	0.249	2.471	0.016^*^
VB9	0.007	0.009	0.086	0.771	0.444
SAM	0.001	3.82 × 10^−4^	0.354	3.336	0.001^**^
Hcy	−1.212 × 10^−4^	4.69 × 10^−5^	−0.250	−2.583	0.012^**^

MMSE, Mini-Mental State Examination; MoCA, Montreal Cognitive Assessment; VB2, vitamins b2; VB9, vitamins b9 or folate; SAM, S-adenosylmethionine; Hcy, homocysteine. ^*^P < 0.05 and ^**^P < 0.01.

**Table 5:** Linear Regression for MOCA.

**Model**	**Unstandardized**	**Standard error**	**Standardized**	***t***	***p***
**M**_1_					
(Intercept)	41.857	7.012		5.969	<0.001
Age	−0.328	0.102	−0.322	−3.226	0.002^**^
Education	0.154	0.109	0.139	1.411	0.163
VB2	0.092	0.045	0.212	2.045	0.045^*^
VB9	0.016	0.013	0.137	1.188	0.239
SAM	0.002	5.445 × 10^−4^	0.317	2.908	0.005^**^
Hcy	−1.305 × 10^−4^	6.688 × 10^−5^	−0.195	−1.951	0.055

MMSE, Mini-Mental State Examination; MoCA, Montreal Cognitive Assessment; VB2, vitamins b2; VB9, vitamins b9, or folate; SAM, S-adenosylmethionine; Hcy, homocysteine. ^*^P < 0.05 and ^**^P < 0.01.

## 4 Discussion

The present study aimed to examine the associations between ARCI and levels of folate, vitamin B, SAM and Hcy. The findings revealed a high prevalence of cognitive impairment among older adults, with high levels of VB2, folate and SAM being strongly associated with better cognitive function in this population. Logistic regression suggested that education and Hcy levels were independently associated with the likelihood of cognitive impairment. Multiple linear regression analyses demonstrated that cognitive function scores (MMSE and MoCA) significantly declined with increasing age. Conversely, higher levels of VB2 and SAM were associated with significant improvements in cognitive function scores, whereas elevated Hcy levels were associated with decreased MMSE and MoCA scores. These results suggest that folate metabolic profiles are significantly associated with the presence of ARCI in older adults.

Aging is the strongest known risk factor for developing dementia, with the risk increasing significantly with age, particularly among individuals aged 65 years and older (22). The present study aligns with this finding, as we found a higher prevalence of cognitive impairment and lower cognitive function scores with advancing age. Our research found that lower education levels were significantly associated with an increased risk of cognitive impairment, which is consistent with previous studies suggesting that higher education levels may be associated with a protective effect (23) and contribute to greater “cognitive reserve” (24).

Several epidemiological studies have examined associations between folate metabolic profiles, which are believed to play a role in maintaining central nervous system function, and cognitive function or dementia (25). For example, higher intake of VB2 has been shown to improve cognitive performance across multiple domains in middle-aged and older adults (26) and is associated with better cognitive function in the elderly. Additionally, higher MoCA scores have been linked to elevated serum levels of VB9, VB6, and VB12 (27). Vitamin B supplementation has been found to reduce serum Hcy and plasma SAH levels while increasing plasma SAM levels and may lead to improvements in MoCA scores, particularly in naming and orientation (28). Hyperhomocysteinemia is recognized as a risk factor for cognitive decline, mild cognitive impairment, and Alzheimer's disease (29). Moreover, Hcy, folate, and VB12 levels have been associated with the degree of cognitive impairment in older adults (30, 31). Our study supports previous findings, confirming the positive correlation between VB2, VB9, and SAM levels and cognitive function, as well as the negative correlation between Hcy levels and cognitive function. However, not all studies have found consistent or linear associations. For instance, Ding et al. (32) reported inverse U-shaped relationships between folate or B12 status and cognitive scores in older adults, with no statistically significant associations observed across much of the biomarker distribution, and no evidence of interaction between the two vitamins.

Despite this, VB2, SAM, and Hcy were also significantly associated with the presence of age-related cognitive impairment in older adults. To better understand these associations, it is important to consider the underlying biochemical pathways through which folate-related metabolites influence neural function.

VB2, in its coenzyme forms flavin mononucleotide (FMN) and flavin adenine dinucleotide (FAD), is involved in numerous enzymatic reactions, including the conversion of tryptophan to nicotinic acid. A deficiency in VB2 decreases the efficiency of these reactions, thereby affecting neurotransmitter synthesis (33). Folate, in its active form 5-methyltetrahydrofolate, provides methyl groups that are transferred to Hcy to generate methionine, which is subsequently converted to SAM through an ATP-dependent reaction. SAM, as a methyl donor, plays a critical role in the methylation of DNA, RNA and proteins (11). These methylation processes are essential for regulating gene expression, DNA repair, and maintaining genome stability. Deficiencies in VB9 or SAM led to hypomethylation and impact nervous system function; however, these mechanisms remain hypothetical and require further investigation. Therefore, low levels of VB2, VB9, and SAM may be involved in neurological dysfunction by reducing DNA methylation and neurotransmitter synthesis, ultimately inducing cognitive decline.

In addition, elevated levels of Hcy, a neurotoxic substance, have been suggested to be associated with cognitive deficits through several proposed mechanisms, including activating the N-methyl-D-aspartate (NMDA) receptor (34) or through conversion to homocysteine thiolactone, leading to neuronal damage and apoptosis (35). While these pathways are well-supported by prior studies, in our cross-sectional data we observed an association between elevated Hcy and ARCI, which may reflect similar neurotoxic processes in this population. These findings suggest that maintaining optimal levels of folate, vitamin B and SAM may be associated with better cognitive health and a lower likelihood of ARCI.

In the present study, multiple linear regression showed that VB2 and SAM were significantly positively correlated with cognitive function scores and Hcy was negatively correlated. Logistic regression further indicated that Hcy was independently associated with a higher likelihood of cognitive impairment. This result may reflect that higher levels of VB2 and SAM are associated with better cognitive performance, possibly due to their roles in neuroprotection and metabolic support, although they may not be significantly associated with a lower likelihood of cognitive impairment. On the contrary, elevated Hcy levels may exert stronger effects once exceeding certain thresholds, potentially contributing more directly to the presence of impairment, as reflected in the logistic model. These observations highlight the possibility of differential associations between folate-related metabolites and various dimensions of cognitive health.

Our study also identified a significant difference in the baseline characteristics of total and direct bilirubin levels between the two groups of patients. It has been shown that gut microbial metabolites are strongly associated with cognitive function, especially the potential regulatory role of bile acid metabolism in neurological health (36–38). Building on this observation, we propose to further investigate the potential interplay between folate metabolic profiles and bile acid metabolic profiles in relation to cognitive function. This line of inquiry may help elucidate the complex mechanisms of the gut-brain axis in the regulation of cognitive function and could contribute to identifying candidate biomarkers for future research.

## 5 Limitation

While this study identifies significant associations between folate metabolic profiles and ARCI, several limitations must be acknowledged. The cross-sectional design precludes establishing causality, as the temporal relationship between folate metabolism biomarkers and cognitive outcomes remains unclear, and reverse causation cannot be ruled out. Additionally, due to financial and time constraints, the study was limited to a relatively small sample of elderly patients from a specific region, which may restrict the generalizability of findings to broader populations. The recruitment of participants from a hospital-based setting, rather than a community-based cohort, may introduce selection bias, as these individuals may differ systematically from the general older adult population. Furthermore, the lack of detailed data on dietary habits and vitamin supplement intake, which relied partly on self-reports, may have influenced the accuracy of folate metabolic profiles. Potential confounders, including physical activity, comorbidities, and medication use, were not fully controlled, potentially affecting the observed associations.

To address these limitations, future research should prioritize large-scale, prospective cohort studies involving diverse populations to enhance generalizability and clarify causal relationships. Comprehensive assessments of dietary habits, lifestyle factors, and repeated biomarker measurements over time would provide deeper insights into the dynamic interplay between folate metabolism and ARCI, enabling the development of targeted interventions.

## 6 Conclusion

In general, this study identified significant associations between VB2, SAM, Hcy levels and cognitive function in older adults, suggesting that folate metabolic profiles may serve as informative indicators of age-related cognitive status. These findings provide a foundation for future research into metabolic biomarkers of cognitive health by underscoring the importance of early metabolic screening in aging populations and its potential role in shaping preventive strategies for cognitive decline.

## Data Availability

The raw data supporting the conclusions of this article will be made available by the authors, without undue reservation.
